# MRI-detection rate and incidence of lumbar bleeding sources in 190 patients with non-aneurysmal SAH

**DOI:** 10.1371/journal.pone.0174734

**Published:** 2017-04-03

**Authors:** Sepide Kashefiolasl, Nina Brawanski, Johannes Platz, Markus Bruder, Christian Senft, Gerhard Marquardt, Volker Seifert, Stephanie Tritt, Juergen Konczalla

**Affiliations:** 1 Department of Neurosurgery, Goethe-University, Frankfurt am Main, Germany; 2 Institute of Neuroradiology, Goethe-University, Frankfurt am Main, Germany; Universitatsklinikum Freiburg, GERMANY

## Abstract

**Background:**

Up to 15% of all spontaneous subarachnoid hemorrhages (SAH) have a non-aneurysmal SAH (NASAH). The evaluation of SAH patients with negative digital subtraction angiography (DSA) is sometimes a diagnostic challenge. Our goal in this study was to reassess the yield of standard MR-imaging of the complete spinal axis to rule out spinal bleeding sources in patients with NASAH.

**Methods:**

We retrospectively analyzed the spinal MRI findings in 190 patients with spontaneous NASAH, containing perimesencephalic (PM) and non-perimesencephalic (NPM) SAH, diagnosed by computer tomography (CT) and/or lumbar puncture (LP), and negative 2nd DSA.

**Results:**

190 NASAH patients were included in the study, divided into PM-SAH (n = 87; 46%) and NPM-SAH (n = 103; 54%). Overall, 23 (22%) patients had a CT negative SAH, diagnosed by positive LP. MR-imaging of the spinal axis detected two patients with lumbar ependymoma (n = 2; 1,05%). Both patients complained of radicular sciatic pain. The detection rate raised up to 25%, if only patients with radicular sciatic pain received an MRI.

**Conclusion:**

Routine radiological investigation of the complete spinal axis in NASAH patients is expensive and can not be recommended for standard procedure. However, patients with clinical signs of low-back/sciatic pain should be worked up for a spinal pathology.

## Introduction

Subarachnoid hemorrhage (SAH) is normally due to intracranial lesions, mostly aneurysms or vascular malformations. In up to 15% of all spontaneous subarachnoid hemorrhages, there is no evidence of an intracranial vascular pathology [1]. Recently published data showed increasing numbers of patients with non-aneurysmal SAH (NASAH) [2]. The evaluation of patients with subarachnoid hemorrhage with negative digital subtraction angiography (DSA) is therefore important and furthermore sometimes a diagnostic challenge.

Among patients suffering from SAH, non-aneurysmal (perimesencephalic (PM) and non-perimesencephalic (NPM)) SAH seems to have better prognosis compared to aneurysm related SAH. Nonetheless, patients with a NPM-SAH have an increased risk of a worse neurological outcome and should be monitored closely [3,4]. Additional investigations to definitively detect an intracranial cause of the hemorrhage and to assess the course of the disease, treatment options and prognosis, such as repeat digital subtraction angiography (DSA) or CT angiography (CT-A), are especially recommended in NPM-SAH patients [5–9].

The causes of NASAH are potentially diverse, and the exact pathogenic mechanism of bleeding in these cases is often not identified. As possible explanations, vascular malformations, ruptured aneurysms of perforating arteries or small venous anomalies, atypical venous drainage patterns, basilar artery dissection, trauma, influence of antithrombotic medication and a case of a spinal vascular malformation, have been described [2,8,10,11,12,13,14].

Among above-mentioned bleeding sources SAH following trauma is presented with an incidence of 15–40% of severe head injuries [15].

Traumatic spinal subarachnoid hemorrhage (SSAH) is very rare. Most cases occur as a complication of spinal procedures such as spinal anesthesia and are often associated with blood coagulation abnormalities, such as anticoagulation therapy or thrombocytopenia [16].

SAH due to spinal lesions occurs in less than 1% [17] and is mostly a consequence of a spinal tumor or arteriovenous malformation (AVM) [18,19]. There is however little systematic literature data concerning the incidence and type of spinal lesions responsible for SAH.

The aim of this study was to reassess the yield of standard MR-imaging of the complete spinal axis to rule out spinal masses in non traumatic SAH patients without a cranial bleeding source. Additionally, the incidence and detection rate of MRI was calculated using additional important clinical factors and different hemorrhage patterns depending on location of subarachnoid blood on the initial CT-scan differentiating between PM- and NPM- (Fisher Grade: stratified between Fisher 3 and non-Fisher 3 blood pattern) NASAH.

## Materials and methods

### Ethics statement

The retrospective clinical study was approved by the local ethics committee of the Goethe-University, Frankfurt am Main, Germany.

### Patient population

Data of 1404 consecutive patients admitted from 1999 to 2012 with SAH to our Institution were retrospectively analyzed from our prospectively maintained neurovascular database. Patients with cerebral aneurysm or cranial vascular malformation (n = 1214) were excluded from the study.

We investigated the yield of clinical and radiological characteristics in 190 patients with subsequent diagnosis of spontaneous NASAH with headache as leading symptom, based on hemorrhage pattern on computer tomography (CT), or a positive lumbar puncture (LP), and no identifiable intracranial vascular pathology on cerebral digital subtraction angiography (DSA). All patients were stratified into PM-SAH and NPM-SAH (including patients with CT-negative/LP-positive SAH; see below for definition criteria) and registered for assessment of the complete spinal axis by standard MR-imaging (1,5 Tesla, and since 2004 3 Tesla MRI; see below for the MRI protocol) during their hospital stay.

### Clinical and diagnostic management

According to a standardized SAH protocol according to international guidelines [17] all patients with SAH, diagnosed by SAH pattern, or confirmed by lumbar puncture, underwent cerebral digital subtraction angiography (DSA) to rule out intracranial vascular bleeding sources. Patients in whom the bleeding source was detected to be an aneurysm or vascular malformation were excluded from the study and treated by surgical or endovascular aneurysm occlusion based on an interdisciplinary consensus [2]. If absence of intracranial vascular pathology was confirmed by a neuroradiologist, patients received a 2^nd^ angiography regularly approximately 14 days to 60 days after the ictus. The timing of repeat DSA is still debatable. This was demonstrated in the study by Dalyai et al., in which the first repeat DSA after 7 days revealed 10 aneurysms, but a second repeat DSA after 6 weeks revealed another 7 patients with a vascular abnormality [6].

Nevertheless, it is difficult to draw any conclusions, as only a few studies have focused on the timing of repeat DSA. Given the vasospasm period and the chance of a thrombosed aneurysm, it is reasonable to perform a repeat DSA at least 10–14 days after the ictus [20].

All patients without selective risk factors also received a cerebral/cervical MRI and additionally undergo MR-imaging of the complete spinal axis to detect a spinal bleeding source. Standard spinal MRI protocol included T2- and non-enhanced/ contrast enhanced T1- weighted images in biplanar orientation.

A PM-SAH was defined as the presence of subarachnoid blood in the cisterns around the midbrain with the center of the bleeding immediately anterior to the midbrain, with or without extension to the ambient and chiasmatic cisterns, horizontal part of the sylvian fissure and posterior horns of the lateral ventricles. Extensive intraventricular hemorrhage and intraparenchymal hemorrhage were exclusive of PM-SAH [21]. Patients with an aneurysmal SAH pattern, described as blood located in the interpeduncular cistern, as well as in the Sylvian cistern, interhemispheric cistern and convexity, or a negative computer tomography with a positive lumbar puncture, and negative digital subtraction angiography (DSA), were classified as NPM-SAH.

All patients were monitored for at least 14 days to rule out any delayed complications such as neurological deficits followed by cerebral vasospasm, vasospasm-based cerebral infarction, hydrocephalus and re-bleeding.

### Statistical analysis

Data analyses were performed using the computer software package (IBM SPSS, version 22, IBM SPSS Inc.; Armonk, NY). Normal distributed variables were expressed as mean values with standard deviations (SD).

## Results

### Patient characteristics

Among 1404 patients suffering from non-traumatic SAH, who were admitted to the Department of Neurosurgery of Goethe University Hospital between 1999–2012, 1201 patients had an aneurysmal SAH in the first DSA and were excluded of the study.

Second DSA ruled out a bleeding source in 13 patients: anterior circulation aneurysms (n = 5); posterior circulation aneurysms (n = 5); AVM (n = 2); AV-fistula (n = 1).

After diagnostic workup, NASAH was diagnosed in one hundred ninety cases of 1404 SAH patients (14%). The mean age was 55,8 years and 40% was female.

Baseline characteristics were compared between PM and NPM-SAH. The initial CT investigation showed a PM-SAH pattern in 46% (n = 87). A NPM-SAH pattern was determined in 103 patients (54%). 23 NPM-SAH patients (22%) presented with negative computed tomographic scan were diagnosed by lumbar puncture.

Routine magnetic resonance imaging of the spinal axis was performed in patients with NASAH without any medical limitation factors. MRI findings were lumbar ependymoma in two patients (1,05%) (Table 1). Overall, 99% of the patients (102/103) suffering from NPM-SAH complained of severe headache and 8% (n = 8) of low-back/sciatic pain. According to the distribution of subarachnoid blood 66% (n = 68) had a non-Fisher 3 pattern SAH (including Fisher 1, 2 and 4).

**Table 1:** Patient characteristics and radiological assessment.

Patient characteristics	NASAH	PM-SAH	NPM-SAH
DSA negativ			
Number of patients	190	87 (46% of NASAH)	103 (54% of NASAH)
Age (years)	55,8 ± 15	51,5 ± 13	60 ± 16
Female	77 (40%)	31 (36%)	46 (45%)
Low-back/leg pain			
Yes	10 (5%)	2 (2%)	8 (8%)
No	180 (95%)	85 (98%)	95 (92%)
Fisher -Score			
Fisher 3 pattern	35 (18%)	0 (0%)	35 (34%)
non-Fisher 3 pattern	155 (82%)	87 (100%)	68 (66%)
Fisher 1			23 (34%)
Fisher 2			45 (66%)
Fisher 4			0 (0%)
CT-negative (diagnosis by LP)	23 (12%)	0 (0%)	23 (22%)
Positive MR spinal axis	2 (1%)	0 (0%)	2 (2%)

Patient characteristics and radiological assessment of 190 non-aneurysmal subarachnoid hemorrhage patients.

Data are shown as n (%), or median ± standard deviation; PM-SAH perimesencephalic subarachnoid hemorrhage, NPM-SAH non-perimesencephalic subarachnoid hemorrhage

Both patients with lumbar ependymoma complained of headache and low-back/sciatic pain, and had a CT negative NPM-SAH on the initial CT scan with a xanthochromic cerebrospinal fluid (CSF) in the lumbar puncture. In both cases the ependymomas were located in the lumbar region. Both patients underwent resection of the spinal mass and histological diagnosis proved the presence of ependymomas (Table 2).

**Table 2:** Summary of cases with SAH due to spinal ependymomas.

Patient no.	Gender	Age (y)	Location	Symptoms	Cranial CT for blood	Lumbar puncture	Cerebral DSA
1	M	37	L 1–2 intradural	headache vomiting low-back pain	negative	xanthochromic CFS	negative
2	M	24	L 1–2 intradural	headache dysaesthesia low-back/sciatic pain	negative	xanthochromic CFS	negative

**Fig 1:**
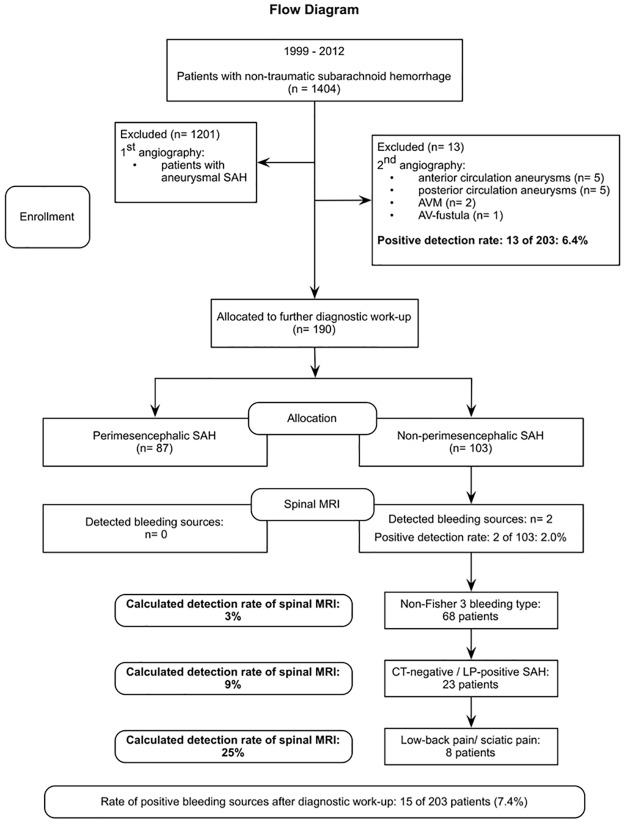
Flow diagram. Flow diagram with detailed information about enrollment, allocation and detection rate.

According to our results, significant predictive criteria for finding a spinal origin of NPM-SAH are low-back/sciatic pain. Considering our achieved statistically data, the expected detection rate of a spinal origin in MRI of patients with NPM-SAH rises up due to following predictive factors: headache (n = 102; 2%), non-Fisher 3 pattern (n = 68; 3%), CT-negative SAH (n = 23; 9%) and low-back/sciatic pain (n = 8; 25%) (Fig 1).

### Case illustration

#### Patient 1

One of the two patients was a 37-year-old male, who complained of severe headache with a typical sudden onset. He also reported nausea, vomiting, and pain in both legs for a few days, before being referred to our department for further diagnosis and treatment. His mental status was normal. Neurological examination showed positive nuchal meningeal symptoms, and bilateral positive Lasegue sign. Acute SAH (Hunt & Hess grade II) was suspected and the performed computer tomography of the head was negative for blood. A lumbar puncture was positive proving xanthochromic CSF, positive lactate and abnormally high level of protein. DSA was performed without detection of bleeding source.

**Fig 2:**
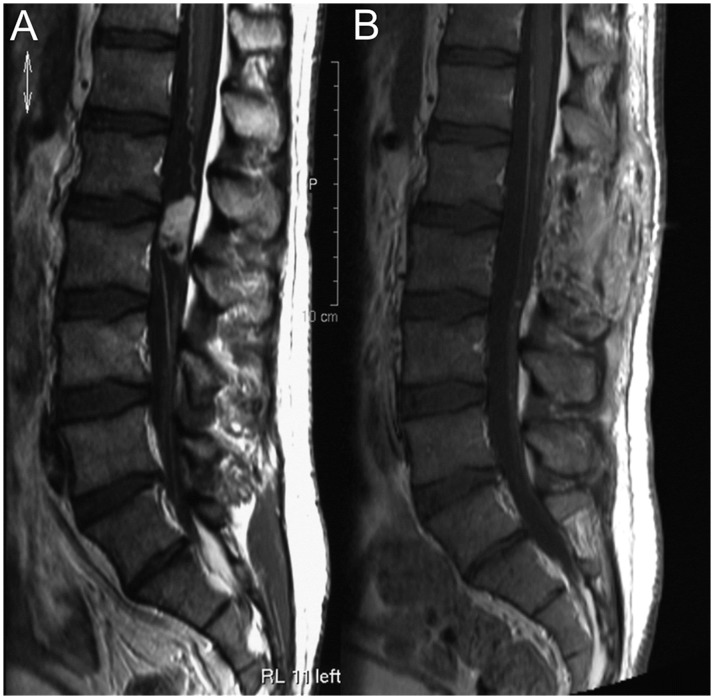
Case illustration of patient number 1. **A** Contrast enhanced T1-weighted images of the lumbar spine in sagittal orientation showed an intradural, extramedullary mass with heterogenous contrast enhancement at the level of L1-2. **B** Postsurgical lumbar MRI revealed total resection of the spinal tumor, but small lumbar and sacral lesions remained unchanged and were suspicious for drop metastasis.

MRI of the spinal axis showed a 3 cm intradural extramedullary mass at the level of L1-2 and further small lumbar and sacral lesions (Fig 2). Surgical resection of the tumor of the filum terminale was performed and the histopathological examination of the specimen showed a hemorrhagic mixopapillary ependymoma (WHO grade I) [22].

#### Patient 2

A-24-year-old man was admitted from another neurological department with suspected diagnosis of a SAH with ictus one week before. He presented severe progressive therapy-resistant headache starting from the neck and continuing "like a cold lightning" into the head, pain like "holes in the head" never suffered before since one week as the first symptom at onset, neck tensions, fever, severe cold, constant sickness, difficulties with equilibrium, eye pain during days, laboratory-defined infect constellation, formication (a tingling sensation under the skin) of the upper limbs, and in addition complained of leg pain on the left side. Three weeks prior to admission, patient has received the diagnosis of type 1 diabetes mellitus. Nine weeks previously, he underwent a laparoscopic knee operation under spinal anesthesia. He did not notice any motor or coordination impairment. Cognitive assessment was unremarkable; cranial nerves, motor and sensory examination was normal, except for a bilateral dysesthesia of the upper limbs caused by hyperventilation produced during a phase of anxiety and panic attack.

Subacute SAH (Hunt & Hess grade I) was diagnosed by positive lumbar puncture with xanthochromic CSF, proof of siderophages, and high abnormally level of lactate, ferritin and protein in CSF. The cerebral angiography was negative.

**Fig 3:**
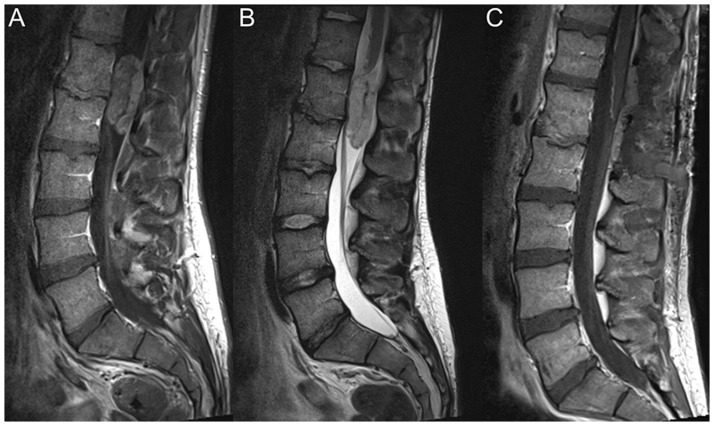
Case illustration of patient number 2. **A** Sagittal T1-weighted gadolinium-enhanced and **B** T2-weighted MRI of the lumbar spine showed a contrast enhancing intradural, extramedullary, T2w hyperintense tumor at the level of L1-2. **C** The tumor was completely removed in the spinal MRI after surgery.

MRI of the spinal axis showed on the L1-2 level an intradural extramedullary tumor of the filum terminale, considered as the source of hemorrhage (Fig 3). The patient underwent complete resection of the spinal mass. The histological examination of the tissue confirmed the diagnosis of a hemorrhagic mixopapillary ependymoma (WHO grade I).

## Discussion

The vast majority of patients with SAH are found to have intracranial lesions, most often vascular pathology like aneurysms or arteriovenous malformations, and in other cases brain tumors, vasculitis or secondary to infarction [17]. Missing intracranial bleeding source is obvious in about 15% of all spontaneous subarachnoid hemorrhages [4]. SAH due to spinal cord masses like ependymomas, nerve sheet tumors, paragangliomas, hemangioblastomas, metastases or meningiomas, however, is a very rare entity. Low-back/sciatic pain and severe headache are often the leading clinical features [22]. In relation to SAH of spinal origin, ependymoma is the most frequent (60%) pathology [18].

A literature review to investigate a spinal cause for NASAH from 1958 to 2006 presented 17 patients with spinal ependymoma and typical clinical features of SAH [22]. Since then, there are following case reports about 2 patients with spinal ependymoma as bleeding source [23,24]. But most data have only small series of patients. Systematic reviews searching for a spinal origin of the hemorrhage, including both vascular malformations and tumors, are missing. Available studies do not present a reliable incidence for a spinal origin as bleeding source in case of NASAH and do not differentiate between different intracranial hemorrhage patterns [11,12,25,26], PM versus NPM (Fisher 3 versus non-Fisher 3) blood pattern, as an important diagnostic aspect.

Another differential diagnosis to explain a symptom complex including negative CT-scan, positive lumbar puncture, negative vascular bleeding sources and typical clinical signs such as low/back pain is delayed intraspinal hemorrhage, which may occur in patients with traumatic brain injury. In particular, patients who have traumatic intracranial SAH or subdural hematoma (SDH) and present delayed pain or neurological deficits should be evaluated for spinal SAH or spinal SDH promptly, even when the patients had no history of direct spinal injury and had no apparent symptoms related to the spinal injury in the initial period of trauma [27].

Due to economical restrictions and/or expedient use of MRI nowadays not every patient receives further diagnostic work-up [28]. This retrospective study assessed the yield of MR-imaging of the complete spinal axis in 190 NASAH patients. The incidence of lumbar ependymoma was 1,05%. Both patients presented no hemorrhage on initial CT scans, positive lumbar punctures, negative angiograms, and low-back/sciatic pain.

In this study, the benefit of spinal imaging in the context of a SAH with negative cerebral angiography was only proved for a small subgroup of NASAH patients suffered from back pain/radicular symptoms. This aspect is therefore socioeconomically of great interest.

In four other studies, the incidence of a spinal origin in NASAH patients, including tumor or vascular malformation, was estimated to be in between 0,05 and 1%, but most data were assessed in series of less than 50 patients [12,17,25,26]. Like in our series, in a recent study by Germans et al., a lumbar ependymoma was diagnosed as bleeding source (also with negative CT) in one of 97 patients with NASAH [23].

German et al. published the newest study according yield of spinal imaging in NPM NASAH as a prospective, multicenter study, including 90 NASAH patients detecting a spinal origin of the bleeding in 4% of the patients: a lumbar ependymoma in 1 patient and a cervical cavernous malformation in 2 patients [29]. Although the study tried to concentrate on NASAH as a subgroup to judge the yield and clinical relevance of MRI of the spinal axis, it only concentrates on sex and age as predictive factors for patients suffering NASAH with a spinal origin as bleeding source. However, this study does not categorize further clinical and radiological risk factors as predictive guidelines in the diagnostic process of NASAH patients with a probably spinal origin as bleeding source.

Our patients with a spinal ependymoma as the origin of the hemorrhage were diagnosed by severe headache and a positive lumbar puncture through a corresponding proof of xanthochromic CSF. On the one hand, with a probably small and low pressure hemorrhage from their spinal lesion the SAH might not have caused any intracranial hemorrhage at all; and on the other hand, the absence of intracranial blood on the initial CT could be a result from a small undetectable amount or a washout of SAH caused by performing the CT several hours after the initial onset, particularly in the case of patient no. 2.

Obviously, a positive CT-scan in NASAH-patients is definitely not an excluding criteria for the following work-up to diagnose a spinal bleeding source. But how far MRI of the spinal axis might be significant, could be depending on other symptoms as parameters of an assistance guide. The expected detection rate of a spinal origin in MRI of patients with NPM-SAH rises up due to following parameters: headache (n = 102; 2%), non-Fisher 3 pattern (n = 68; 3%), CT-negative SAH (n = 23; 9%) and low-back/sciatic pain (n = 8; 25%), see also Fig 1.

However, the absence of typical signs of SAH in patient no. 2 suffering of severe headache of progressive character without typical SAH signs, such as photophobia, vomiting, and meningism, has to be discussed as a limitation of our study. The important point, which has to be mentioned is the diagnosis of SAH despite the atypical character of the headache verifying through xanthochromic CSF, leading in combination with other symptoms, low/back pain and radiculopathy in this case, to a symptom oriented diagnostic work-up to detect a spinal origin as bleeding source.

As a standard diagnostic work-up protocol in case of NASAH without bleeding source in the first and second DSA, cerebral MRI, and non-specific clinical symptoms as guidelines, MRI of the spinal axis should certainly be performed to prove/exclude other possible bleeding sources. However, our study is a trial to create directives for diagnosis- and symptom-orientated work-up for selective radiological detection and to reduce the duration of hospital stay.

According to the clinical relevance of the findings in patients with CT-negative NPM-SAH, diagnosed by a positive lumbar puncture, and accompanying symptoms, such as low-back pain and radiculopathy, a work-up for spinal axis, beginning with lumbar spine, should be considered as a further investigation for SAH.

## Conclusion

SAH caused by spinal pathology is very rare. According to this study, lumbar ependymoma has an incidence of 1,05% in NASAH. Routine radiological investigation of the spinal axis in every NASAH patient is therefore not recommended and should be done symptom-orientated. However, MR-imaging of the complete spinal axis in patients with a CT-negative NPM-SAH, positive lumbar puncture and leading clinical symptoms such as low-back/sciatic pain can be useful and reasonable to detect this rare entity of SAH.

## Supporting information

S1 Table Supplemental database. (XLSX)

## References

[1] van Gijn J, Rinkel GJ. Subarachnoid haemorrhage: diagnosis, causes and management. Brain. 2001; 124: 249–278. 11157554 10.1093/brain/124.2.249

[2] Konczalla J, Kashefiolasl S, Brawanski N, Senft C, Seifert V, Platz J. Increasing numbers of non-aneurysmal subarachnoid hemorrhage in the last fifteen years—antithrombotic medication as reason and prognostic factor? J Neurosurg. 2016; 124(6):1731–7. 10.3171/2015.5.JNS15161 26566212

[3] Konczalla J, Platz J, Schuss P, Vatter H, Seifert V, Güresir E. Non-aneurysmal non-traumatic subarachnoid hemorrhage: patient characteristics, clinical outcome and prognostic factors based on a single-center experience in 125 patients. BMC Neurol. 2014; 14:140–147. 10.1186/1471-2377-14-140 24986457 PMC4088361

[4] Konczalla J, Schuss P, Platz J, Vatter H, Seifert V, Güresir E. Clinical outcome and prognostic factors of patients with angiogram-negative and non-perimesencephalic subarachnoid hemorrhage: benign prognosis like perimesencephalic SAH or same risk as aneurysmal SAH? Neurosurg Rev. 2015; 38: 121–127. 10.1007/s10143-014-0568-0 25183063

[5] Agid R, Andersson T, Almqvist H, Willinsky RA, Lee SK, terBrugge KG, et al Negative CT angiography findings in patients with spontaneous subarachnoid hemorrhage: when is digital subtraction angiography still needed? AJNR Am JNeuroradiol. 2010; 31: 696–705. 19942709 10.3174/ajnr.A1884 PMC7964209

[6] Dalyai R, Chalouhi N, Theofanis T, Jabbour PM, Dumont AS, Gonzalez LF, et al Subarachnoid hemorrhage with negative initial catheter angiography: a review of 254 cases evaluating patient clinical outcome and efficacy of short- and long-term repeat angiography. Neurosurgery. 2013; 72: 646–652. 10.1227/NEU.0b013e3182846de8 23277373

[7] Jung JY, Kim YB, Lee JW, Huh SK, Lee KC. Spontaneous subarachnoid haemorrhage with negative initial angiography: a review of 143 cases. J Clin Neurosci. 2006; 13: 1011–101. 10.1016/j.jocn.2005.09.007 16931020

[8] Khan AA, Smith JD, Kirkman MA, Robertson FJ, Wong K, Dott C, et al Angiogram negative subarachnoid haemorrhage: outcomes and the role of repeat angiography. Clin Neurol Neurosurg. 2013; 115: 1470–1475. 10.1016/j.clineuro.2013.02.002 23485251

[9] Konczalla J, Schmitz J, Kashefiolasl S, Senft C, Seifert V, Platz J. Non-aneurysmal subarachnoid hemorrhage in 173 patients: prospective study of long-term outcome. Eur J Neurol. 2015; 22(10):1329–36. 10.1111/ene.12762 26130053

[10] Beseoglu K, Pannes S, Steiger HJ, Hanggi D. Long-term outcome and quality of life after nonaneurysmal subarachnoid hemorrhage. Acta Neurochir (Wien). 2010; 152: 409–416. 19784546 10.1007/s00701-009-0518-8

[11] Fassett DR; Rammos SK, Patel P, Parikh H, Couldwell WT. Intracranial subarachnoid hemorrhage resulting from cervical spine dural arteriovenous fistulas: literature review and case presentation. Neurosurg Focus. 2009. 10.3171/FOC.2009.26.1.E4 19119890

[12] Germans MR, Pennings FA, Sprengers ME, Vandertop WP. Spinal vascular malformations in non-perimesencephalic subarachnoid hemorrhage. J Neurol. 2008; 255: 1910–1915. 10.1007/s00415-009-0021-4 19159064

[13] Schievink WI, Wijdicks EF. Pretruncal subarachnoid hemorrhage: an anatomically correct description of the perimesencephalic subarachnoid hemorrhage. Stroke. 1997; 28: 2572 9412654

[14] Wijdicks EF, Schievink WI. Perimesencephalic nonaneurysmal subarachnoid hemorrhage: first hint of a cause? Neurology. 1997; 49: 634–636. 9270619 10.1212/wnl.49.2.634

[15] Cairns CJ, Finfer SR, Harrington TJ, Cook R. Papaverine angioplasty to treat cerebral vasospasm following traumatic subarachnoid haemorrhage. Anaesth Intensive care. 2003; 31:87–91. 12635402 10.1177/0310057X0303100117

[16] Chen HC, Hsu PW, Tzaan WC. "Migration" of traumatic subarachnoid hematoma? A case report. Surg Neurol. 2008; 70(2):213–6. 10.1016/j.surneu.2007.04.003 17720228

[17] Walton JN. Subarachnoid haemorrhage of unusual aetiology. Neurology. 1953; 3: 517–543. 13063667 10.1212/wnl.3.7.517

[18] Cervoni L, Franco C, Celli P, Fortuna A. Spinal tumors and subarachnoid haemorrhage: pathogenetic and diagnostic aspects in 5 cases. Neurosurg Rev. 1995; 18: 159–162. 8570061 10.1007/BF00383718

[19] Cummings TM, Johnson MH. Neurofibroma manifested by spinal subarachnoid haemorrhage. Am J Roentgenol. 1994; 162: 959–960. 8141025 10.2214/ajr.162.4.8141025

[20] Bakker NB, Groen RJM, Foumani M, Boogaart MU, Eshghi OS, Metzemaekers JMD, et al Repeat digital subtraction angiography after a negative baseline assessment in nonperimesencephalic subarachnoid hemorrhage: a pooled data meta-analysis. A systematic review. J Neurosurg. 2014; 120:99–103. 10.3171/2013.9.JNS131337 24160474

[21] Rinkel GJ, Wijdicks EF, Vermeulen M, Ramos LM, Tanghe HL, Hasan D, et al Nonaneurysmal perimesencephalic subarachnoid hemorrhage: CT and MR patterns that differ from aneurysmal rupture. AJNR Am J Neuroradiol. 1991; 12: 829–834. 1950905 PMC8333493

[22] Ulrich CT, Beck J, Seifert V, Marquardt G. Ependymoma of conus medullaris presenting as subarachnoid haemorrhage. Acta Neurochirurgica. 2008; 150: 185–188. 10.1007/s00701-007-1407-7 18058061

[23] Germans MR, Coert BA, Majoie CBLM, van den Berg R, Verbaan D, Vandertop WP. Spinal axis imaging in non-aneurysmal subarachnoid hemorrhage: a prospective cohort study. J Neurol. 2014; 261: 2199–2203. 10.1007/s00415-014-7480-y 25182702

[24] Nicastro N, Schnider A, Leemann B. Anaplastic medullary ependymoma presenting as subarachnoid hemorrhage. Case Rep Neurol Med. 2013; 2013: 701820 10.1155/2013/701820 23533857 PMC3606746

[25] Little AS, Garrett M, Germain R, Farhataziz N, Albuquerque FC, McDougall CG, et al Evaluation of patients with spontaneous subarachnoid hemorrhage and negative angiography. Neurosurg. 2007; 61: 1139–1150. 10.1227/01.neu.0000306091.30517.e7 18162892

[26] van Beijnum J, Straver DC, Rinkel GJ, Klijn CJ. Spinal arteriovenous shunts presenting as intracranial subarachnoid haemorrhage. J Neurol. 2007; 254: 1044–1051. 10.1007/s00415-006-0485-4 17401739 PMC2779417

[27] Kim TJ, Koh EJ, Choe KT. Spinal Subarachnoid Hemorrhage Migrated from Traumatic Intracranial Subarachnoid Hemorrhage. Korean J Neurotrauma. 2016; 12(2): 159–162. 10.13004/kjnt.2016.12.2.159 27857928 PMC5110909

[28] Kalra VB, Wu X, Matouk CC, Malhotra A. Use of follow-up imaging in isolated perimesencephalic subarachnoid hemorrhage: a meta-analysis. Stroke. 2015; 46(2):401–6. 10.1161/STROKEAHA.114.007370 25523050

[29] Germans MR, Coert BA, Majoie CBLM, van den Berg R, Lycklama à Nijeholt G, Rinkel GJE, et al Yield of spinal imaging in nonaneurysmal, nonperimesencephalic subarachnoid hemorrhage. Neurology. 2015; 84;1337–1340. 10.1212/WNL.0000000000001423 25724231

